# Time-Restricted Feeding Reduces the Detrimental Effects of a High-Fat Diet, Possibly by Modulating the Circadian Rhythm of Hepatic Lipid Metabolism and Gut Microbiota

**DOI:** 10.3389/fnut.2020.596285

**Published:** 2020-12-01

**Authors:** Yuqian Ye, Haopeng Xu, Zhibo Xie, Lun Wang, Yuning Sun, Huayu Yang, Dandan Hu, Yilei Mao

**Affiliations:** ^1^Department of Liver Surgery, Peking Union Medical College (PUMC) Hospital, PUMC & Chinese Academy of Medical Sciences (CAMS), Beijing, China; ^2^Department of Hepatobiliary Surgery, Sun Yat-Sen University Cancer Center, Guangzhou, China

**Keywords:** time-restricted feeding, circadian rhythm, high-fat diet, gut microbiota, intermittent fasting

## Abstract

**Background:** Time-restricted feeding, also known as intermittent fasting, can confer various beneficial effects, especially protecting against obesity, and related metabolic disorders, but little is known about the underlying mechanisms. Therefore, the present study aims to investigate the effects of time-restricted feeding on the circadian rhythm of gut microbiota and hepatic metabolism.

**Methods:** Eight-week-old male Kunming mice received either a normal diet *ad libitum*, a *high-fat* diet *ad libitum*, or a high-fat diet restricted to an 8-h temporal window per day for an experimental period of 8 weeks. Weight gain and calorie intake were measured weekly. Serum metabolites, hepatic sections and lipid metabolites, gut microbiota, and the hepatic expression of *Per1, Cry1, Bmal1*, SIRT1, SREBP, and PPARα were measured at the end of the experimental period. The composition of gut microbiota and the expression of hepatic genes were compared between four timepoints.

**Results:** Mice that received a time-restricted high-fat diet had less weight gain, milder liver steatosis, and lower hepatic levels of triglycerides than mice that received a high-fat diet *ad libitum* (*p* < 0.05). The numbers of *Bacteroidetes* and *Firmicutes* differed between mice that received a time-restricted high-fat diet and mice that received a high-fat diet *ad libitum* (*p* < 0.05). Mice fed a time-restricted high-fat diet showed distinct circadian rhythms of hepatic expression of SIRT1, SREBP, and PPARα compared with mice fed a normal diet *ad libitum*, as well as the circadian rhythm of the abundance of *Bacteroidetes* and *Firmicutes*.

**Conclusions:** Time-restricted feeding is associated with better metabolic conditions, perhaps owing to alterations in gut microbiota and the circadian pattern of molecules related to hepatic lipid metabolism, which were first to report.

## Background

Time-restricted feeding (TRF), in some cases also referred as intermittent fasting, is a feeding pattern where all nutrient intake is restricted to certain hours of the day with no limitation on nutrient quality or quantity (1, 2). Studies in as early as 1980s proposed that reducing food availability protected against aging and improved life span in mice (3, 4). Recent researches as well as our studies in rodent animals concerning TRF are increasingly revealing its various beneficial effects, including preventing diet-induced obesity and its associated metabolic disorders (1, 5–8), preventing inflammatory bowel diseases and colorectal cancer and neurodegenerative disorders (9, 10), and alleviating hepatic ischemia reperfusion injury (11). The rhythm of feeding can greatly influence the development of liver steatosis and remodel the hepatic circadian metabolome, which suggests a potential mechanism of TRF (12, 13). By restricting food availability to a certain temporal window at the same time of every day, TRF can trigger a food-anticipatory activity depending on the endogenous circadian clock (14).

In mammals, the intrinsic circadian system is a complicated feedback network that maintains and regulates proper rhythms in metabolic pathways required for organism homeostasis (15, 16). Mammalian circadian clock contains a central oscillator located in the suprachiasmatic nuclei (SCN) of the hypothalamus and various peripheral tissue, with harmonious bidirectional interactions (17–19). The molecular bedrock of core circadian clock is composed of a series of diurnally fluctuated proteins, and among which the transcription factors CLOCK, BMAL1 (Brain and muscle Arnt-like protein-1), Cryptochrome (*Cry1 and Cry2*), and Period (*Per1, Per2, and Per3*) act as the core molecules (20, 21). In peripheral organs including liver, skeletal muscle, and adipose tissue, similar circadian rhythms also occur with feeding/fasting acting as the primary regulator (16, 18, 22). The core circadian molecule composed of CLOCK:BMAL1 complex is closely connected to cellular metabolism by regulating the cyclic transcription of *Nampt* (nicotinamide phosphoribosyl transferase) and directing the cyclic synthesis of NAD^±^ (23, 24). SIRT1 (Sirtuin1), as an NAD^±^-dependent enzyme, is regulated in this manner and functions as an energy sensor by responding to the cellular levels of metabolic intermediates such as NAD^±^ and NADH (25). Previous studies found that SIRT1 also deacetylates transcription factors that regulate the choice of oxidative or glycolytic metabolic strategy and fatty acid synthesis, namely PPARα (peroxisome proliferator-activated receptor) and SREBP (sterol regulatory element-binding protein) (26, 27).

The desynchronization of normal circadian rhythm is associated with various pathological conditions, among which obesity and related metabolic disorders have become a concerned issue (28). The circadian desynchronization can be implemented using a homozygous CLOCK mutation model, which presents attenuated diurnal rhythm of feeding behavior and obesity (29). On the other side, diet-induced obesity (DIO) also leads to changes in the rhythmicity of core circadian clock proteins. In DIO mice, the expression of CLOCK and BMAL1 was altered associated with other metabolic disorders like leptin resistance and adiponectin deficiency (30, 31). In addition, DIO generates comprehensive rhythmic changes in the expression of proteins related to fatty acid synthesis and oxidation, especially SREBP and PPARα (32, 33). Gut microbiota analysis also reveals changes in DIO mice, characterized by a reduction in the *Bacteroidetes* phylum and an increase in the *Firmicutes* phylum (34). A few previous studies also tested the circadian rhythmicity of gut microbiota, and suggested distinct differences between normal mice and DIO mice (35–38).

The prevention and treatment for DIO has arose wide discussion, among which TRF was also intensely studied as a non-pharmaceutical intervention. It has been shown that TRF, by merely changing the external rhythm of feeding, can protect against obesity, inflammation and hepatic steatosis (5, 7, 39). However, the underlying mechanism of TRF for improving DIO-related disorders is not completely understood. It is reported that TRF can alter the hepatic expression level of core circadian proteins and mRNAs at certain timepoints of the day, resulting in changes in the circadian rhythmicity (13). And gut microbiota is reported to be another possible mechanism, as TRF decreases *Firmicutes* abundance and increases *Bacteroidetes* abundance (36, 40). In the present study, we subjected mice to TRF regimen of a high-fat diet for 8 h per day and explored the regulatory effects of TRF on weight gain and the circadian rhythmicity of hepatic lipid metabolism and gut microbiota. Our findings provided deeper understanding into the use of TRF as a non-pharmaceutical intervention.

## Methods

### Animals

All animal experiments were carried out in accordance with the guidelines of the Animal Care and Use Committee of the Salk Institute, and permitted by the Animal Welfare Committee of Peking Union Medical College Hospital (approval XHDW-2017-00). A total of sixty male Kunming mice at 8 weeks of age were housed five to a cage under a 12-h light/dark schedule for the experimental period of 8 weeks. All mice were provided by Academy of Military Medical Sciences, Beijing, China.

### Feeding Schedule and Diets

All mice were randomly assigned to three feeding schedules: (1) normal diet-*ad libitum* (NA group), (2) high-fat diet (HFD)-*ad libitum* (FA group), and (3) HFD-TRF (FT group). All mice were fed normal diet before the experimental period. Normal-diet mice were fed a standard chow diet (D12450B; 20% protein, 10% fat, 70% carbohydrates; Research Diets, Inc., New Brunswick, NJ, United States) with unrestricted access. The other groups received HFD (D12492; 20% protein, 59.9% fat, 20.1% carbohydrates; Research Diets, Inc.) with either *ad libitum* access or during an 8-h window between Zeitgeber time (ZT) 16 and ZT24 every day. All mice were maintained on the experimental feeding regimens for 8 weeks and body mass was weighed weekly. Access to food was controlled by providing and removing the diet tray daily. The mass of remaining food was measured and recorded daily for every cage, and average daily calorie intake was calculated by the following formula: (original food mass-remaining food mass) × calorie-mass ratio/total number of mice. The light was turned on at ZT0 and turned off at ZT 12. More details about the diet were available upon request.

### Tissue Collection and Measurement of Liver Metabolites

Twenty mice from each group were sacrificed over a 24-h period at ZT0, ZT8, ZT12, and ZT20 (five mice at each ZT timepoint). Mice fed *ad libitum* were sacrificed after a fasting period to keep in accordance with FT mice. Livers and rectums were collected using laparotomy. Liver lobes were separated and the median lobes were fixed in 4% buffered formalin. Other liver lobes were homogenized to test liver lipid metabolites. Rectal contents were snap-frozen and stored at −80°C prior to analysis.

### Measurement of Serum Metabolites

Blood samples were collected from eight mice from each group at the end of the experimental period (two mice at each ZT timepoint). Samples were snap-frozen and stored at −80°C prior to analysis. Serum metabolites representing liver function parameters and lipid profile were quantified by automatic chemistry analyzer (Chemray 240; Rayto Life and Analytical Sciences Co., Shenzhen, China).

### Liver Morphometric Analysis

Formalin-fixed liver tissues were dehydrated and embedded in optimal cutting temperature compound. Ten-micrometer sections were cut and stained with freshly prepared Oil Red O. Five mice from each group were used for the morphometric evaluation of liver steatosis. The sections were evaluated under light microscopy by two independent pathologists who were blinded to the groupings. Liver steatosis was graded by its magnitude as described previously (41). Briefly, the degree of steatosis was graded by the percentage of the liver section that was occupied by fat vacuoles at 100× magnification in 10 fields for every mouse (grade 0: ≤5%; grade 1: 5–25%; grade 2: 26–50%; grade 3: 51–75%; grade 4: >75%).

### Western Blots

Whole cell protein was extracted from frozen liver tissue, and total protein lysates were subjected to SDS-PAGE on 8–20% acrylamide gels, electro-transferred to polyvinylidene difluoride membranes (MilliporeSigma, Burlington, MA, United States), and probed overnight at 4°C in the presence of the following primary antibodies: anti-SIRT1 (1:1,000, 13161-1-ap, Proteintech Group, Inc., Rosemont, IL, United States), anti-PPARα (1:1,000, ab8934, Abcam, Cambridge, United Kingdom), and anti-SREBP (1:1,000, bs-1402R, Bioss Antibodies, Woburn, MA, United States). For protein detection, we used horseradish peroxidase-conjugated secondary antibodies (Wuhan ServiceBio Technology Co., Wuhan, China). Five samples per group per timepoint were assayed; protein levels were normalized to β-actin (1:3,000, GB12001, Wuhan ServiceBio Technology Co.). Densitometry analysis of protein bands was conducted using the open-source image processing software ImageJ (https://imagej.net/ImageJ).

### RNA Extraction and Quantitative RT-PCR

Total RNA was extracted from liver tissues using a RNeasy mini kit (Qiagen, Hilden, Germany). RNA concentrations were determined by spectrophotometric trace (NanoDrop, Thermo Fisher Scientific, Waltham, MA, United States). Total RNA (200–1,000 ng) was transcribed into cDNA (volume: 20 μL) using a RevertAid First Strand cDNA Synthesis Kit (#K1622, Thermo Fisher Scientific) following the manufacturer's instructions. We then used FastStart Universal SYBR Green Master (04913914001, Roche, Basel, Switzerland) to determine the relative abundance of the mRNAs of interest. The following primers were used: (a) *Per1*; accession number NM_001159367.2, forward 5′-GGCCAGATTGGTGGAGGTTA-3′ and reverse 5′-TCTGACTGCTGCGGGTGAT-3′; (b) *Cry1*; accession number NM_007771.3, forward 5′-CAGGAGGAGAAACTGAGGCACT-3′ and reverse 5′-GCCACAGGAGTTGCCCATAA-3′; (c) *Bmal1*; accession number NM_001243048.1, forward 5′-GGGGAAATACGGGTGAAATCTA-3′ and reverse 5′-CTGAACCATCGACTTCGTAGCG-3′. PCR reactions were performed in a total volume of 25 μL containing 2.5 μL of diluted cDNA or a reference cDNA sample. The PCR conditions were 95°C for 5 min, followed by 40 cycles of 15 s at 95°C and 60 s at 60°C. Cycles were analyzed by StepOnePlus software (ABI, Foster City, CA, United States). The PCR reaction was followed by analysis of the melting curve.

### Gut Microbiota Analysis

Fecal contents of rectal samples were collected at ZT0, ZT8, ZT12, and ZT20 and preserved in −80°C refrigerator before analysis. Fecal samples were sent to Shanghai Majorbio (Shanghai, China) for 16S rRNA gene V3–V4 sequencing using a MiSeq platform (Illumina, San Diego, CA, USA). The primers used were forward 5′-ACTCCTACGGGAGGCAGCAG-3′ and reverse 5′- GGACTACHVGGGTWTCTAAT-3′. The full report and statistical analysis from Second Genome are available upon request. The original contributions presented in the study are publicly available. This data is available via SRA with BioProject number PRJNA673908.

### Statistical Analysis

All statistical analyses were performed in SPSS version 24.0 (IBM SPSS, Armonk, NY, United States). The data were first subjected to normality tests. For data passing these tests, the differences between groups and differences between different ZT timepoints within same feeding regimen were analyzed by one-way ANOVA followed by the Bonferroni multiple comparison test. The differences between ZT timepoints were used to evaluate the circadian rhythmicity. Analyses were two-sided. Quantitative, continuous data are expressed as the mean ± SEM in the figures, unless noted otherwise. *p* < 0.05 was considered statistically significant.

## Results

### TRF Prevents HFD-Induced Weight Gain and Improves Serum Lipid Profile

**Figure 1:**
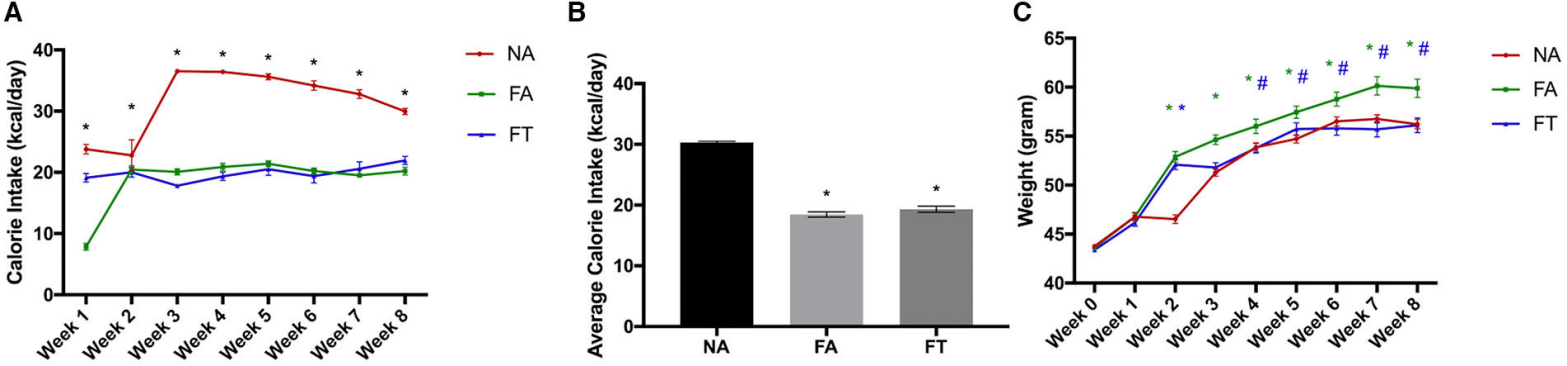
Effects of feeding regimen on calorie intake and body weight. Food intake and body weights were measured weekly. **(A)** Calorie intake for every week; **(B)** Average calorie intake for the 8 weeks; **(C)** Body weights at every week. Data were shown as mean ± SEM (*n* = 20 for each group). Data were analyzed by one-way ANOVA followed by Bonferroni multiple comparison test. In **(A)** *FA vs. NA, *p* < 0.05 and FT vs. NA, *p* < 0.05. In **(B)**, compared to the NA group, *p < 0.05. In **(C)**, compared to the NA group, *p < 0.05 (green and blue asterisks represent for FA and FT group, respectively), compared to the FA group, #*p* < 0.05. NA, mice fed a normal diet *ad libitum*; FA, mice fed a high-fat diet *ad libitum*; FT, mice fed a time-restricted high-fat diet.

Eight-week-old male wildtype Kunming mice were fed a normal diet *ad libitum*, HFD *ad libitum*, or HFD with TRF for an experimental period of 8 weeks. As shown in Figure 1, FA mice consumed less calories than NA mice (18.46 ± 0.98 vs. 30.3 ± 0.47 kcal/day, *p* < 0.001) but gained more weight (16.25 ± 5.80 vs. 12.47 ± 2.61 g, *p* = 0.003) during the experimental period. Compared to FA mice, FT mice consumed similar amounts of calories (19.33 ± 1.11 kcal/day, *p* = 0.335) but gained less weight (12.72 ± 5.04 g, *p* = 0.006).

We then examined the effects of feeding regimen on serum metabolites. Serum levels of total cholesterol (TC), triglycerides (TG), and low-density lipoprotein cholesterol (LDL-C) were different between groups (see Additional File S1). Compared to NA mice, FA mice had a higher level of LDL-C and comparable serum levels of TC and TG. FT mice had a lower level of TG (0.54 ± 0.09 mmol/L) compared to both NA (0.82 ± 0.05 mmol/L, *p* = 0.017) and FA mice (0.77 ± 0.28 mmol/L, *p* = 0.036). And no significant differences were observed between groups regarding levels of albumin, alanine aminotransferase or bilirubin.

### TRF Alleviates Liver Steatosis and Decreases Hepatic TG Level

**Figure 2:**
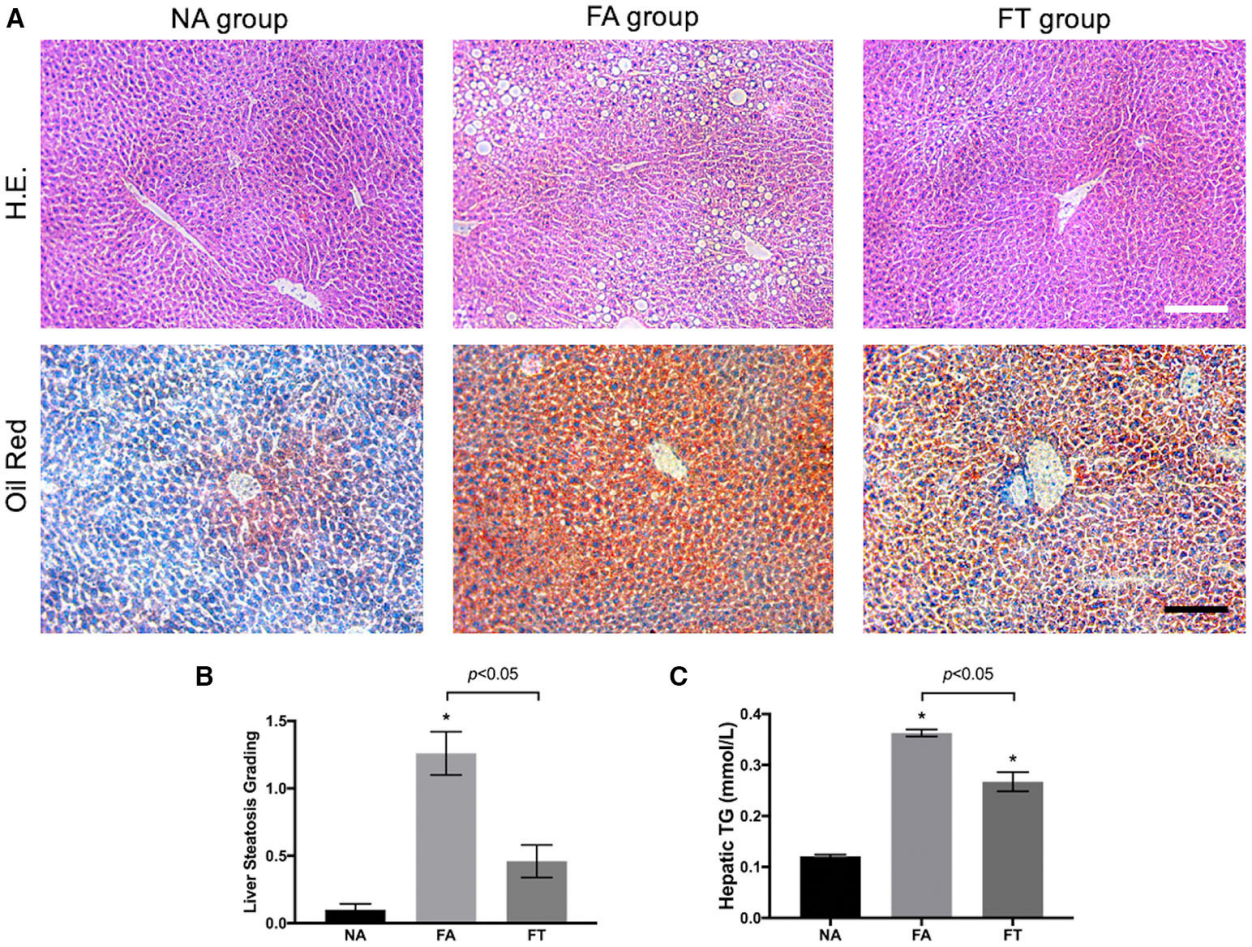
Effects of feeding regimen on liver steatosis. **(A)** Representative images of hematoxylin and eosin-stained and Oil Red O-stained liver tissue sections from three experimental groups. Slides were photographed at a magnification of 200×. Scale Bar = 50 μm. **(B)** Liver steatosis grading (*n* = 5). **(C)** Hepatic TG levels (*n* = 20). Data were shown as mean ± SEM. Data were analyzed using one-way ANOVA followed by Bonferroni multiple comparison test. Compared to NA group, *p < 0.01. NA, mice fed a normal diet *ad libitum*; FA, mice fed a high-fat diet *ad libitum*; FT, mice fed a time-restricted high-fat diet; TG, triglyceride.

We then investigated whether feeding regimens influenced the severity of liver steatosis. Median lobes of the liver were formalin-fixed and sectioned to assess liver steatosis. As shown in Figure 2A, FA mice had a large numbers of fat vacuoles in the liver section in contrast with NA and FT mice. Liver steatosis was graded in 5 mice from each group (Figure 2B). FA mice had a higher grading of liver steatosis than NA mice, and FT mice had a lower grading of liver steatosis than FA mice while comparable to NA mice.

We then tested hepatic lipid levels using liver homogenates at different ZT timepoints. Compared to NA mice, both FA mice and FT mice had higher overall hepatic levels of TG, TC, LDL-C, and HDL-C (Figure 2C and Additional File S2). FT mice had a lower hepatic TG level than FA mice (0.267 ± 0.084 vs. 0.363 ± 0.031 mmol/L, *p* = 0.011). The overall hepatic levels of other lipid metabolites of FT mice were comparable to FA mice.

Despite changes in the overall hepatic TG level, FA mice, and FT mice did not exhibit significant fluctuations among different ZT timepoints (see Additional File S3). The differences of the four tested hepatic lipid levels between ZT timepoints were not significant in any group, except for the hepatic LDL-C level of NA mice (*p* = 0.030). The hepatic TG levels of FA mice were higher than NA mice at all four ZT timepoints. And FT mice had intermediate hepatic TG levels compared to both NA and FA mice at all four timepoints, while the differences between FT and NA mice were not significant.

### TRF Changes Gut Microbiota Composition at Certain ZT Timepoints

**Figure 3:**
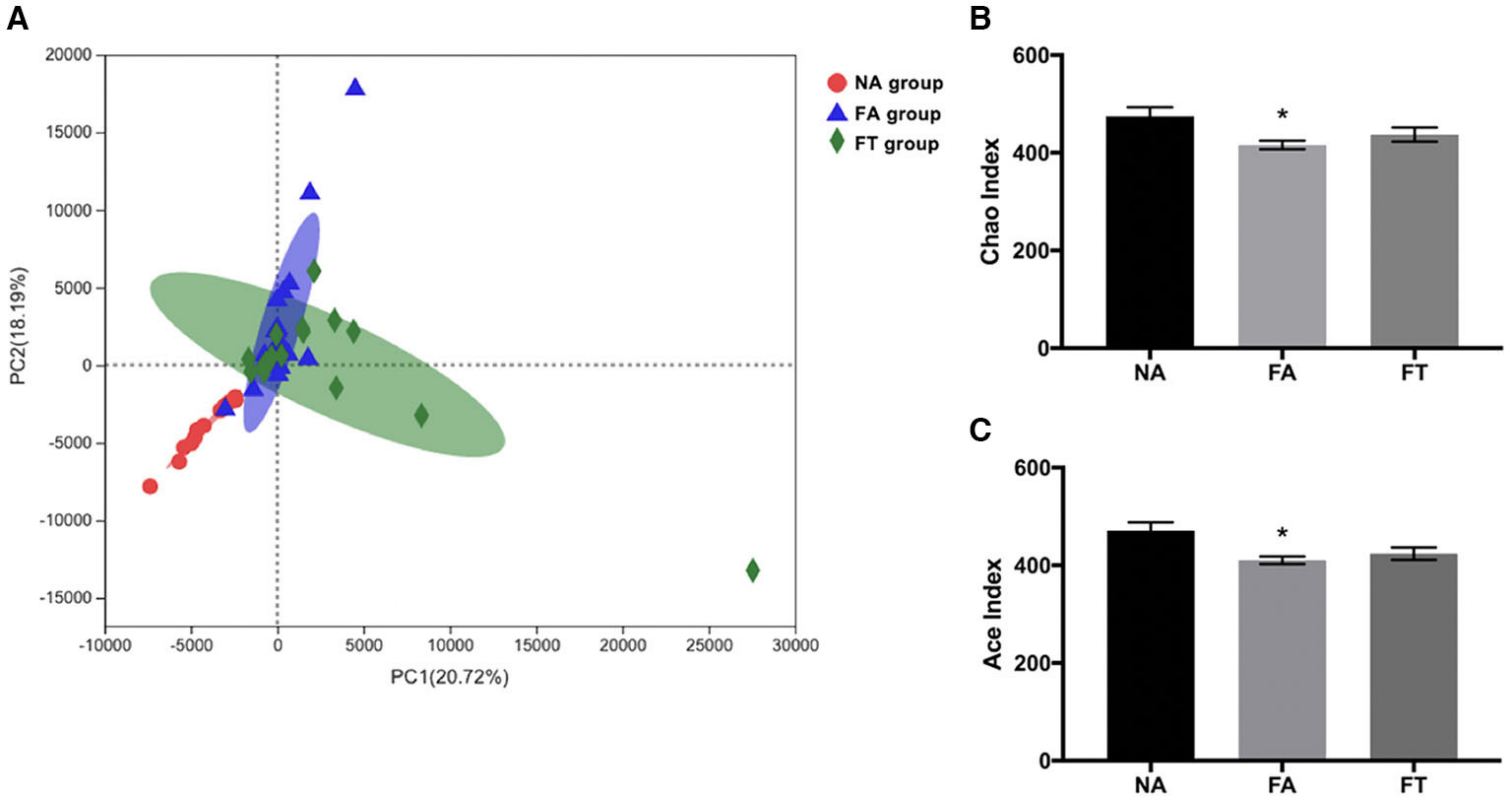
The effects of feeding regimen on the diversity of gut microbiota. **(A)** Principal component analysis (PCA) of beta diversity on OTU level. **(B)** Alpha diversity by Chao index. **(C)** Alpha diversity by ACE index. Data were shown as mean ± SEM (*n* = 16–20 for each group). Indexes were compared using one-way ANOVA followed by Bonferroni multiple comparison test. Compared to NA group, *p < 0.05. NA, mice fed a normal diet *ad libitum*; FA, mice fed a high-fat diet *ad libitum*; FT, mice fed a time-restricted high-fat diet; OTU, operational taxonomic unit.

To further investigate how feeding regimen influences serum and hepatic lipid profiles, we tested the composition and richness of gut microbiota from mice of all three groups at different ZT timepoints. Fecal samples were collected and genomic DNA was extracted from fecal samples and sequenced to identify various bacterial taxa. A total of 672 filtered operational taxonomic units (OTUs) were detected. Principal component analysis (PCA) of bacteria demonstrated a separation between NA group and the other two groups, while FA group and FT group were close to each other (Figure 3A). Sample richness was compared between groups using Chao and Ace indexes (Figures 3B,C). Compared to NA mice, FA mice had a lower Chao index (*p* = 0.013) and Ace index (*p* = 0.006). And the differences between FT mice and NA mice were not significant, regarding both Chao index (*p* = 0.190) and Ace index (*p* = 0.073).

**Figure 4:**
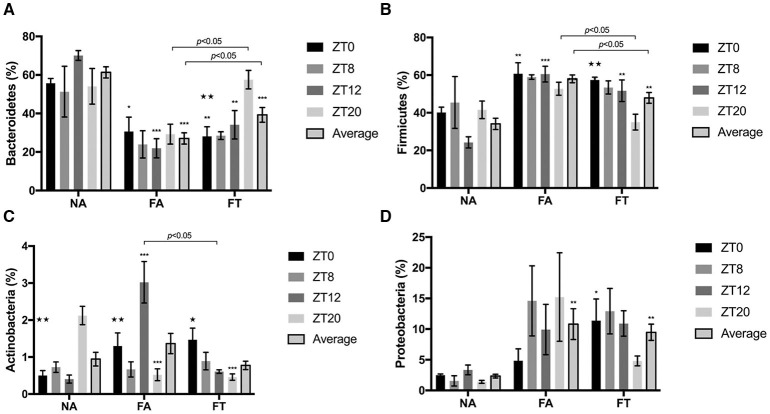
The effects of feeding regimen on gut microbiome composition at different ZT timepoints. Four predominant phyla were presented: **(A)** *Bacteroidetes*, **(B)** *Firmicutes*, **(C)** *Actinobacteria*, and **(D)** *Proteobacteria*. Relative abundance levels of phyla were shown as mean ± SEM (%) (*n* = 4–5 for each timepoint; *n* = 18–20 for the average). Data were compared between groups at same ZT timepoints using one-way ANOVA followed by Bonferroni multiple comparison test. Compared to NA group, *p < 0.05, **p < 0.01, ***p < 0.001. Data were also compared between different ZT timepoints within same feeding regimen using one-way ANOVA, ^⋆^*p* < 0.05, ^⋆⋆^*p* < 0.01. NA, mice fed a normal diet *ad libitum*; FA, mice fed a high-fat diet *ad libitum*; FT, mice fed a time-restricted high-fat diet; ZT, Zeitgeber time.

The composition of gut microbiota is also different between groups (Figure 4). Compared to NA mice, FA mice had a higher relative abundance of *Firmicutes* (58.04 ± 9.33% vs. 34.10 ± 13.49%, *p* < 0.001) and *Proteobacteria* (10.81 ± 11.23% vs. 2.34 ± 1.38%, *p* = 0.002), and a lower relative abundance of *Bacteroidetes* (27.02 ± 13.06% vs. 61.34 ± 12.99%, *p* < 0.001). And FT mice had an intermediate relative abundance of *Firmicutes* (47.89 ± 12.86%) and *Bacteroidetes* (39.28 ± 17.08%) compared to NA and FA mice (see Additional File S4).

To investigate whether the composition of gut microbiota is circadian rhythmically dependent, data were compared between different ZT timepoints (Figure 4). The abundance of *Actinobacteria* was diurnally fluctuated in all three groups, and the abundance levels of *Bacteroidetes* and *Firmicutes* were diurnally fluctuated (*p* = 0.003 and 0.006, respectively), in FT mice (Figures 4A–C). Compared to NA mice, both FA and FT mice had a lower *Bacteroidetes* abundance level and a higher *Firmicutes* abundance level at ZT0 and ZT12, and the differences at ZT8 and ZT20 were not significant (Figures 4A,B). Compared to FA mice, FT mice had a higher *Bacteroidetes* abundance level at ZT20 (57.58 ± 10.77% vs. 29.27 ± 11.56%, *p* = 0.029) and a lower *Firmicutes* level at ZT20 (35.04 ± 9.38% vs. 52.77 ± 7.73%, *p* = 0.026). And FT mice had a slightly higher *Bacteroidetes* abundance level and lower *Firmicutes* abundance level at other timepoints compared to FA mice, but the differences were not significant. The abundance levels of several subphylum categories were analyzed and compared between ZT timepoints (see Additional File S5). The relative abundance of family *Lachnospiraceae* showed oscillation between timepoints in NA and FT mice (*p* = 0.010 and 0.045, respectively), but not in FA mice (*p* = 0.2769). Abundance levels of family *Ruminococcacea* also showed a circadian rhythm in NA and FT mice (*p* = 0.012 and 0.037, respectively), while no rhythm in FA mice (*p* = 0.249).

### TRF Alters the Circadian Expression of Proteins Related to Lipid Metabolism in Liver

**Figure 5:**
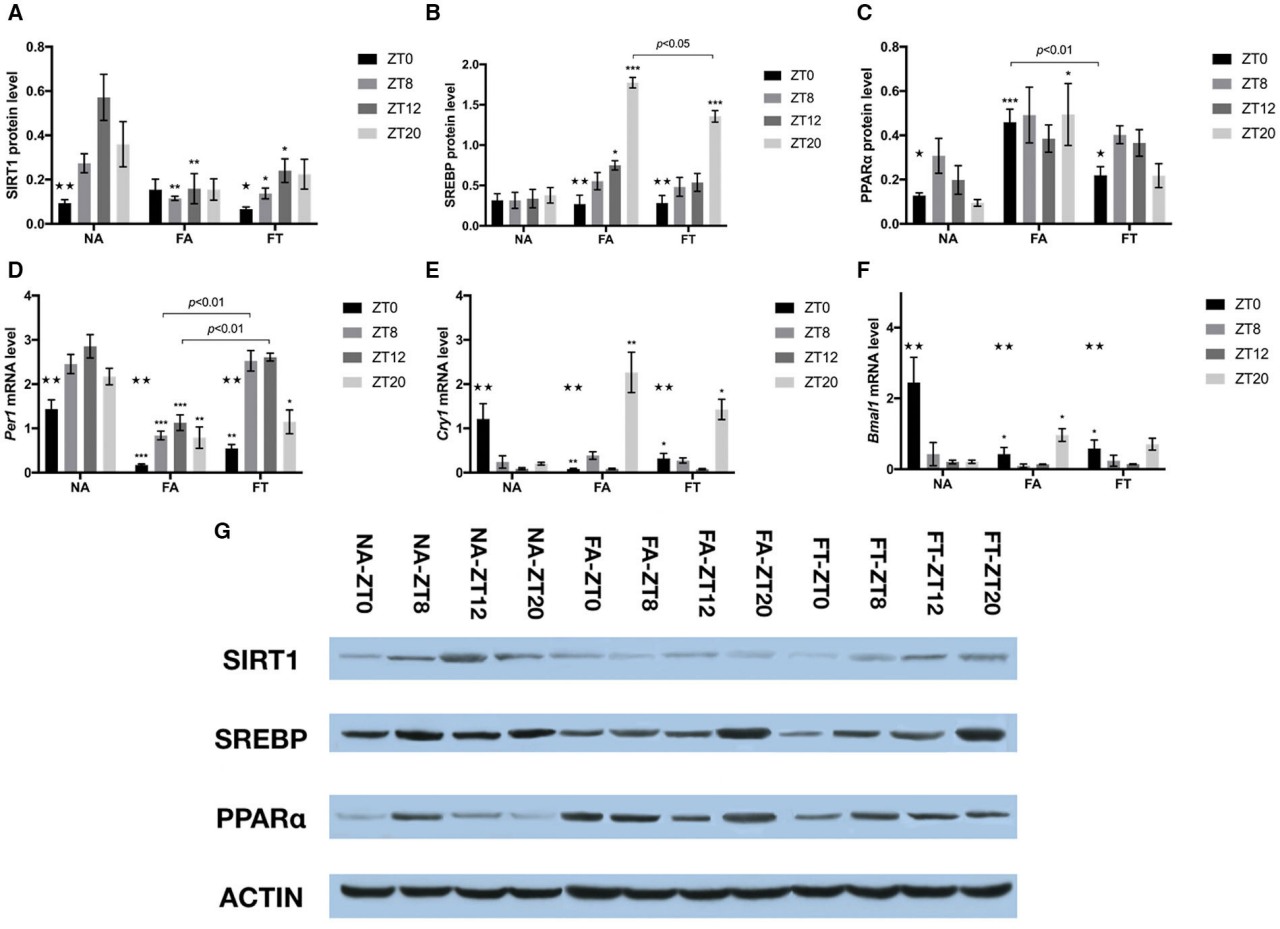
The effects of feeding regimen on hepatic circadian genes. Hepatic levels of proteins **(A–C,G)** and mRNAs **(D–F)** related to circadian rhythm or energy metabolism. Livers were isolated by laparotomy in each group, and the whole cell protein levels of SIRT1, SREBP, and PPARα were measured by Western blotting: **(A)** SIRT1, Sirtuin1; **(B)** SREBP, Sterol Regulatory Element Binding Protein; **(C)** PPARα, Peroxisome Proliferator-activated Receptor α. Relative mRNA expressions were tested using real-time qPCR: **(D)** *Per1* mRNA; **(E)** *Cry1* mRNA; **(F)** *Bmal1* mRNA. Relative amounts of proteins (relative to β-actin) or mRNAs were shown as mean ± SEM. **(G)** The representative image of immunoblotting analysis of lysates from liver tissue. Data were shown as mean ± SEM (*n* = 4–5 for each timepoint). Data were compared between groups at same ZT timepoints using one-way ANOVA followed by Bonferroni multiple comparison test. Compared to NA group, *p < 0.05, **p < 0.01, ***p < 0.001. Data were also compared between different ZT timepoints within same feeding regimen using one-way ANOVA, ^⋆^*p* < 0.05, ^⋆⋆^*p* < 0.01. FA, mice fed a high-fat diet *ad libitum*; FT, mice fed a time-restricted high-fat diet; NA, mice fed a normal diet *ad libitum*; ZT, Zeitgeber time.

In order to investigate whether feeding regimens affect the hepatic expression of proteins involved in energy and lipid metabolism, we tested the whole cell protein levels of SIRT1, SREBP, and PPARα (Figures 5A–C,G). In NA mice, the hepatic protein levels of SIRT1 and PPARα exhibited clear circadian rhythms (*p* = 0.004 and 0.048, respectively), while the protein levels of SREBP were comparable between different timepoints. In NA mice, the circadian rhythm of SIRT1 expression manifested as peaking at ZT12 and reaching bottom at ZT0, with a peak-to-valley ratio of 6.08. Such rhythm disappeared in FA mice (*p* = 0.906) and was restored in FT mice (*p* = 0.049), with a peak-to-valley ratio of 3.61. Regarding differences between groups, however, FT mice had comparable cellular protein levels of SIRT1 compared to FA mice at ZT8 and ZT12, both of which were lower than NA mice (Figure 5A). Both FA and FT mice exhibited clear circadian rhythms of SREBP expression (*p* < 0.001 for both groups, Figure 5B). The rhythms of FA mice and FT mice were similar, presented as peaking at ZT20 and reaching bottom at ZT0, with peak-to-valley ratios of 6.58 and 4.78, respectively. Regarding group comparisons, FA mice had a higher SREBP amount at ZT12 (*p* = 0.033) while FT mice had a comparable SREBP amount (*p* = 0.511) compared to NA mice. Both FT and FA mice had higher amounts of SREBP at ZT20 compared to NA mice, while that of FT mice was significantly lower than FA mice (*p* = 0.008). The expression of PPARα in NA mice peaked at ZT8 and reached bottom at ZT20, with a peak-to-valley ratio of 3.25 (Figure 5C). Such circadian rhythm was also observed in FT mice but not in FA mice. Regarding group comparisons, cellular amounts of PPARα were elevated FA mice at ZT0 (*p* < 0.001) and ZT20 (*p* = 0.044) compared to NA mice. Such differences were not observed in FT mice compared to NA mice, and FT mice had a lower amount of PPARα at ZT0 compared to FA mice (*p* = 0.004).

### TRF Mildly Alters the Circadian Expression of Endogenous Core Clock Genes in Liver

To examine whether TRF also affected the expression of endogenous circadian genes, we tested hepatic mRNA amounts of *Per1, Cry1*, and *Bmal1* using real-time qPCR (Figures 5D–F). The expression of *Per1, Cry1*, and *Bmal1* exhibited clear circadian rhythm in all three groups. *Per1* expression peaked at ZT12 and reached bottom at ZT0 in NA mice, with a peak-to-valley ratio of 1.99. *Cry1* and *Bmal1* expression peaked at ZT0 and reached bottom at ZT12 in NA mice, with peak-to-valley ratios of 13.59 and 12.23, respectively. Both FA and FT mice exhibited similar circadian rhythms of *Per1* expression compared to NA mice, peaking at ZT12 and reaching bottom at ZT0, with peak-to-valley ratios of 6.51 and 4.77, respectively (Figure 5D). Regarding group comparisons, *Per1* levels were consistently lower in FA mice compared to NA mice at all four timepoints. Compared to NA mice, FT mice had lower *Per1* levels at ZT0 and ZT20, and comparable *Per1* levels at ZT8 and ZT12, which were higher than FA mice. FA and FT mice presented different circadian rhythms of *Cry1* expression from NA mice, peaking at ZT20, and reaching bottom at ZT12, with peak-to-valley ratios of 26.34 and 18.31, respectively (Figure 5E). Regarding group comparisons, both FA and FT mice had a lower *Cry1* level at ZT0 and a higher *Cry1* level at ZT20 compared to NA mice, while *Cry1* levels were comparable at ZT8 and ZT12. Regarding *Bmal1* level, FA, and FT mice presented different circadian rhythms from NA mice, peaking at ZT20 instead of ZT0, with peak-to-valley ratios of 10.49 and 5.13, respectively (Figure 5F). Compared to NA mice, both FA and FT mice had lower *Bmal1* levels at ZT0, which was the peak of NA mice.

## Discussion

TRF, also known as intermittent fasting, by merely restricting nutrient intake into certain hours of the day with no limitation on nutrient quality or quantity, has been widely reported to not only extend life span, but also protect against various pathological conditions, including HFD-induced obesity and related metabolic disorders (1–5, 42, 43). However, the underlying mechanism of how TRF improves DIO-related disorders is not completely understood. Therefore, the present study aimed to investigate the effects of TRF on the circadian rhythmicity of hepatic lipid metabolism and gut microbiota in mice.

In the present study, under HFD feeding, FA mice showed more weight gain despite less calorie intake, which may be attributed to less increase in the post-prandial metabolic rate (44). FA mice also exhibited significant liver steatosis and elevated hepatic levels of TG compared to NA mice, indicating the HFD-induced obesity mice model was well-established in the study. And implementation of TRF in mice fed HFD protected against obesity and hepatic lipid accumulation, presented with decreased weight gain, severity of liver steatosis, and hepatic TG level compared to FA mice, which was consistent with previous studies in rodent animals (7, 36, 42, 45). Therefore, it was reasonable to further investigate the underlying mechanism of TRF using the well-established HFD-TRF mice model in the present study. And we mainly focused on changes on gut microbiota and hepatic lipid metabolism.

Changes in gut microbiota, characterized by increased *Firmicutes* and *Actinobacteria* abundance and decreased *Bacteroidetes* abundance, as well as absence of daily oscillations in the relative abundance of family *Lachnospiraceae* and *Ruminococcaceae*, have been shown to be associated with DIO in rodent animals (34, 37, 46, 47). Our study reported that TRF resulted in an increase in sample richness, an increase in *Bacteroidetes* abundance and a decrease in *Firmicutes* abundance, which was consistent with previous knowledge. Some studies suggested that the gut microbiota composition oscillated in a diurnal pattern in wildtype mice, though the exact oscillation regularities of specific phyla were not well-studied (35, 36). Our study depicted the oscillation pattern of abundance of *Bacteroidetes, Firmicutes, Actinobacteria*, and *Proteobacteria* using differences between four distinct ZT timepoints and found TRF led to clear oscillation patterns in *Bacteroidetes* and *Firmicutes*. Subphylum analysis on the relative abundance of family *Lachnospiraceae* and *Ruminococcaceae* found significant oscillations in NA mice and FT mice, but not in FA mice, suggesting such circadian rhythms exhibited in NA mice were disturbed by HFD feeding and further restored by TRF. In our study, the circadian rhythm in FT mice is characterized by a prominent increase of *Bacteroidetes* and decrease of *Firmicutes* at ZT20, which was not observed in the other two groups. Considering the feeding window of FT mice was restricted between ZT16 and ZT24, ZT20 basically represented the feeding phase in TRF. Therefore, such oscillation patterns indicated a distinct difference triggered by TRF regimen between feeding and fasting phases.

We then tested the hepatic expression of core circadian clock genes (*Per1, Cry1*, and *Bmal1*) and proteins related to lipid metabolism (SIRT1, SREBP, and PPARα) to further understand the molecular mechanism of TRF. The core circadian feedback circuits composed of CLOCK, BMAL1, PER, and CRY maintain the cell-autonomous circadian rhythm, and further regulate cellular metabolism through intermediate proteins including SIRT1, SREBP, and PPARα (48–53). Previous knowledge regarding the oscillation pattern of mRNA *Per1, Cry1*, and *Bmal1* all manifested clear diurnal rhythms with one peak and one bottom, though the specific patterns were not completely consistent (7, 45, 52, 54). In our study, the circadian rhythmicity in NA mice was comparable to previous studies with similar peak and bottom timepoints (7, 45). Comparison between the three experimental groups showed similar oscillation patterns of *Per1, Cry1*, and *Bmal1* between FT and FA mice though the relative amounts were slightly different, and both FT and FA mice exhibited distinct oscillation patterns for *Cry1* and *Bmal1* compared to NA mice. Therefore, the TRF regimen in our study did not remodel the hepatic core circadian rhythm altered by HFD to a natural rhythmicity, which indicated the core circadian clock was altered by the feeding content rather than feeding schedule.

In peripheral organs, SIRT1 regulates the oscillatory rhythms and metabolic pathways as a metabolic rheostat (51, 52). The present study showed similar circadian expression of SIRT1 in NA and FT mice, but not in FA mice, indicating TRF restored the circadian rhythm of SIRT1 expression. The protein level of SIRT1 was decreased under conditions with fewer calorie intake (FA and FT mice), and it was consistent with previous studies suggesting TRF with calorie restriction can prevent liver lipid accumulation and alleviate liver inflammation by decreasing hepatic SIRT1 level (27, 55, 56). Accumulated evidences showed SIRT1 negatively regulate SREBP and PPAR levels in the liver (27, 57–59). Our study also presented a consistent increase in both SREBP and PPARα regardless of ZT timepoints, which was possibly sequential to the changes of SIRT1. SREBP and PPAR are involved in energy metabolism and liver lipid synthesis and accumulation, and elevations in SREBP and PPAR contribute to the development of obesity (60–63). The hepatic circadian rhythm of PPARα is characterized as peak at ZT8 and bottom at ZT20 in NA mice and the rhythm disappeared in FA mice, which was exactly the situation in our study (64). Our FT mice also exhibited a similar circadian pattern of PPARα with NA mice, suggesting TRF restored the hepatic rhythm of PPARα. Regarding the hepatic rhythm of SREBP, no clear pattern was found in NA mice, while FA mice had a profoundly higher level at ZT20. Though FT mice also exhibited significant differences between ZT timepoints, its SREBP level at ZT20 was lower than that of FA mice. It indicated that TRF reduced the HFD-induced elevation of SREBP at ZT20, but was not able to restore it to a natural condition. Previous studies have suggested the relationship between hepatic metabolism and gut microbiota using fecal microbiota transplantation. In DIO and HFD-TRF mice, changes in the gut microbiota directly altered the metabolism and absorption of fatty acid, which further induced adiposity by stimulating hepatic *de novo* lipogenesis and triglyceride storage through SREBP (46, 65). However, the causal relationship between changes in the gut microbiota and hepatic lipid metabolism, as well as the reasons for body mass changes under TRF condition needs further exploration, where metabolic cages and MR imaging shall be used in further researches.

Herein, our study further demonstrated the beneficial effects of TRF, that TRF altered the average abundance of the gut microflora, as well as the circadian rhythm of proteins related to hepatic lipid metabolism. It suggested this feeding regimen might improve metabolism and restructure circadian rhythms as a non-pharmacological intervention to prevent obesity by modulating the circadian rhythm.

## Conclusions

In general, we demonstrated that TRF had protective effects against DIO-related disorders. It mainly functioned by influencing gut microbiota and hepatic metabolism, especially by restoring the circadian rhythm of gut microbiota composition and hepatic lipid metabolism. These findings provide deeper insight into the use of TRF as a non-pharmacological intervention.

## Data Availability Statement

The raw data supporting the conclusions of this article will be made available by the authors, without undue reservation.

## Ethics Statement

The animal study was reviewed and approved by the Animal Welfare Committee of Peking Union Medical College Hospital.

## Author Contributions

YY, HX, and YM: study conception and design. YM: funding acquisition. ZX, LW, and YS: acquisition of data. YY, DH, and HY: statistical analysis. YY, HX, and DH: analysis and interpretation of data. YY: drafting of manuscript. DH and YM: critical revision. All authors: approved the final version of the article, including the authorship list.

## Conflict of Interest

The authors declare that the research was conducted in the absence of any commercial or financial relationships that could be construed as a potential conflict of interest.

## Supplementary Material

Additional File S1 Effects of feeding regimen on serum metabolites summarizes levels of serum metabolites of the three groups.

Additional File S2 Effects of feeding regimen on hepatic lipid levels summarizes overall hepatic levels of TG, TC, LDL-C, and HDL-C of the three groups.

Additional File S3 Effects of feeding regimens on hepatic lipid levels at different ZT timepoints summarizes hepatic levels of TG, TC, LDL-C, and HDL-C of the three groups at ZT0, ZT8, ZT12, and ZT20. Effects of feeding regimen on hepatic lipid levels. **(A)** Triglycerides (TG). **(B)** Total cholesterol (TC). **(C)** Low-density lipoprotein cholesterol (LDL-C). **(D)** High-density lipoprotein cholesterol (HDL-C). Data were shown as mean ± SEM (*n* = 5 for each column). Data were compared between groups at same ZT timepoints using one-way ANOVA followed by Bonferroni multiple comparison test. Compared to NA group, ^*^*p* < 0.05, ^**^*p* < 0.01. Data were also compared between different ZT timepoints within same feeding regimen using one-way ANOVA, ^⋆^*p* < 0.05. NA, mice fed a normal diet *ad libitum*; FA, mice fed a high-fat diet *ad libitum*; FT, mice fed a time-restricted high-fat diet; ZT, Zeitgeber time.

Additional File S4 Effects of feeding regimen on gut microbiota composition summarizes the abundance levels of 4 predominant phyla in gut microbiota of the three groups.

Additional File S5 Effects of feeding regimen on gut microbiome composition at different ZT timepoints (analyzed on subphylum level). Presents the relative abundance levels of 3 categories of interest. Effects of feeding regimen on gut microbiota. **(A)** family *Lachnospiraceae*. **(B)** family *Ruminococcus*. **(C)** genus *Lactococcus*. Relative abundance levels were shown as mean ± SEM (%) (*n* = 4–5 for each timepoint; *n* = 18–20 for the average). Data were compared between groups at same ZT timepoints using one-way ANOVA followed by Bonferroni multiple comparison test. Compared to NA group, ^*^*p* < 0.05, ^**^*p* < 0.01, ^***^*p* < 0.001. Data were also compared between different ZT timepoints within same feeding regimen using one-way ANOVA, ^⋆^*p* < 0.05. NA, mice fed a normal diet *ad libitum*; FA, mice fed a high-fat diet *ad libitum*; FT, mice fed a time-restricted high-fat diet; ZT, Zeitgeber time.
