# Supplementary material for: Time-Restricted Feeding Reduces the Detrimental Effects of a High-Fat Diet, Possibly by Modulating the Circadian Rhythm of Hepatic Lipid Metabolism and Gut Microbiota
Source: Front Nutr. 2020 Dec 1;7:596285. doi: 10.3389/fnut.2020.596285 (PMC7793950; doi:10.3389/fnut.2020.596285)
Supplement: Additional File S1 — Effects of feeding regimen on serum metabolites summarizes levels of serum metabolites of the three groups. [file Table_1.DOCX]

Additional file 1. Effects of feeding regimen on serum metabolites. (n=8)

|  | TC | TG | HDL-C | LDL-C | albumin | ApoA1 | ApoB | ALT | TBil | DBil |
| --- | --- | --- | --- | --- | --- | --- | --- | --- | --- | --- |
| NA | 3.00±0.25 | 0.82±0.05 | 2.00±0.22 | 0.20±0.07 | 30.9±3.77 | 0.21±0.06 | 0.14±0.02 | 56.5±16.7 | 1.35±0.49 | 0.63±0.25 |
| FA | 3.34±0.38 | 0.77±0.28 | 1.88±0.10 | 0.32±0.06 ^*^ | 30.2±3.05 | 0.22±0.04 | 0.13±0.01 | 70.4±36.6 | 1.59±0.75 | 0.55±0.39 |
| FT | 3.56±0.34 ^*^ | 0.59±0.16 ^*^ | 2.20±0.08 | 0.30±0.08 | 34.5±1.47 | 0.27±0.01 | 0.12±0.01 | 43.1±10.2 | 1.63±0.49 | 0.77±0.31 |
| *p* value | 0.033 | 0.030 | 0.506 | 0.026 | 0.056 | 0.157 | 0.176 | 0.176 | 0.764 | 0.510 |

Data were shown as mean ± SD. n=8 for each group. Data were analyzed using one-way ANOVA followed by Bonferroni multiple comparison test. Compared to the NA group, **p*<0.05.

NA, mice fed a normal diet ad libitum; FA, mice fed a high-fat diet ad libitum; FT, mice fed a time-restricted high-fat diet. TC: total cholesterol; TG: triglycerides; HDL-C: high-density lipoprotein cholesterol; LDL-C: low-density lipoprotein cholesterol; Apo: apolipoprotein; ALT: alanine transaminase; Tbil: total bilirubin; Dbil: direct bilirubin.
