# Supplementary material for: Time-Restricted Feeding Reduces the Detrimental Effects of a High-Fat Diet, Possibly by Modulating the Circadian Rhythm of Hepatic Lipid Metabolism and Gut Microbiota
Source: Front Nutr. 2020 Dec 1;7:596285. doi: 10.3389/fnut.2020.596285 (PMC7793950; doi:10.3389/fnut.2020.596285)
Supplement: Additional File S2 — Effects of feeding regimen on hepatic lipid levels summarizes overall hepatic levels of TG, TC, LDL-C, and HDL-C of the three groups. [file Table_2.DOCX]

Additional file 2. Effects of feeding regimen on hepatic lipid levels. (n=20)

|  | TC | TG | HDL-C | LDL-C |
| --- | --- | --- | --- | --- |
| NA | 0.066±0.009 | 0.121±0.015 | 0.015±0.003 | 0.134±0.016 |
| FA | 0.145±0.032 ^*^ | 0.363±0.031 ^*^ | 0.041±0.013 ^*^ | 0.208±0.036 ^*^ |
| FT | 0.148±0.045 ^*^ | 0.267±0.084 ^*, #^ | 0.042±0.016 ^*^ | 0.201±0.046 ^*^ |
| *p* value | <0.001 | <0.001 | <0.001 | <0.001 |

Data were shown as mean ± SD. n=20 for each group. Data were analyzed using one-way ANOVA followed by Bonferroni multiple comparison test. Compared to the NA group, **p*<0.001; compared to FA group, #*p*<0.05.

NA, mice fed a normal diet ad libitum; FA, mice fed a high-fat diet ad libitum; FT, mice fed a time-restricted high-fat diet. TC: total cholesterol; TG: triglycerides; HDL-C: high-density lipoprotein cholesterol; LDL-C: low-density lipoprotein cholesterol.
