# Supplementary figures and images for: Time-Restricted Feeding Reduces the Detrimental Effects of a High-Fat Diet, Possibly by Modulating the Circadian Rhythm of Hepatic Lipid Metabolism and Gut Microbiota
Source: Front Nutr. 2020 Dec 1;7:596285. doi: 10.3389/fnut.2020.596285 (PMC7793950; doi:10.3389/fnut.2020.596285)

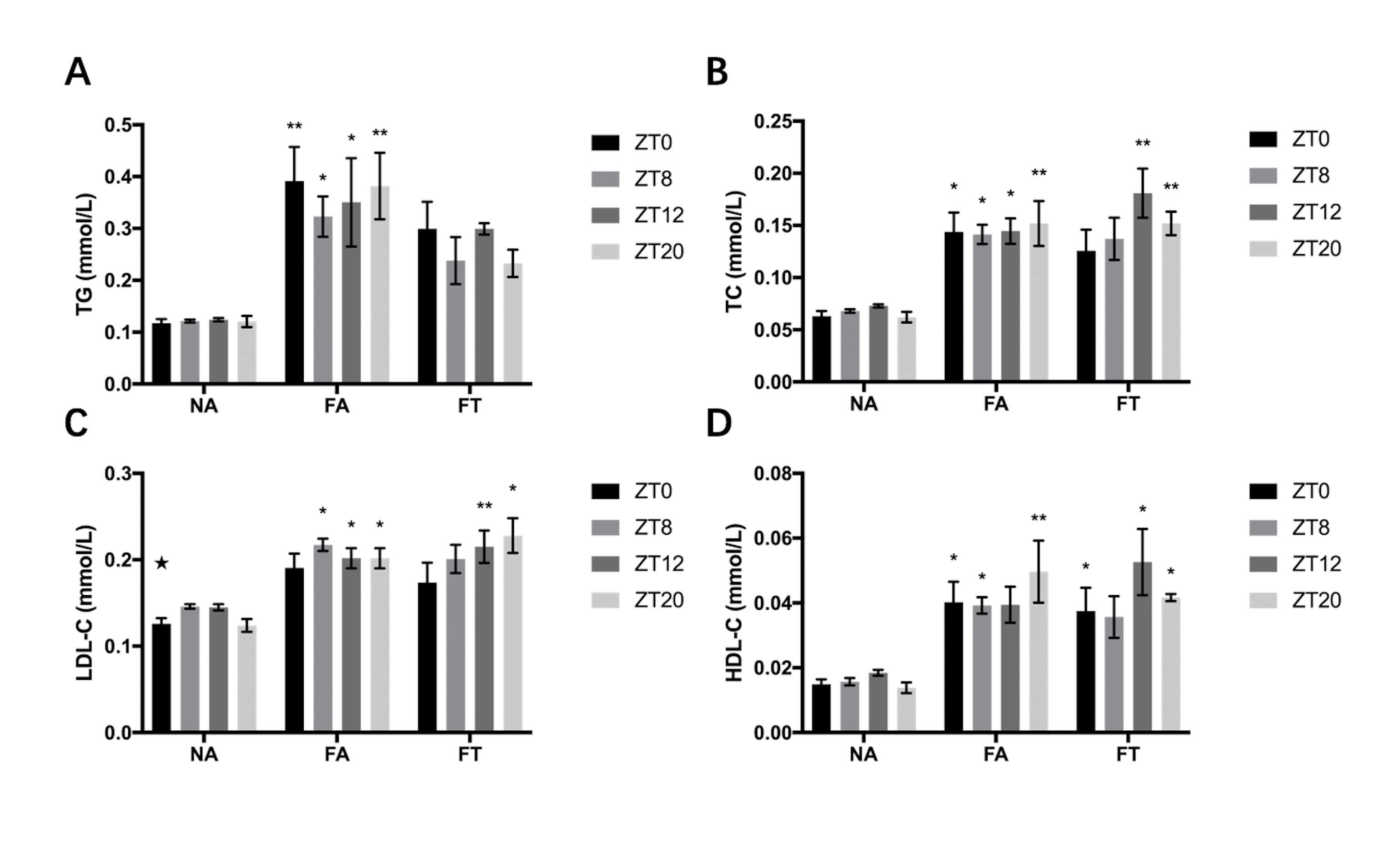

Supplement: Additional File S3 — Effects of feeding regimens on hepatic lipid levels at different ZT timepoints summarizes hepatic levels of TG, TC, LDL-C, and HDL-C of the three groups at ZT0, ZT8, ZT12, and ZT20. Effects of feeding regimen on hepatic lipid levels. (A) Triglycerides (TG). (B) Total cholesterol (TC). (C) Low-density lipoprotein cholesterol (LDL-C). (D) High-density lipoprotein cholesterol (HDL-C). Data were shown as mean ± SEM (n = 5 for each column). Data were compared between groups at same ZT timepoints using one-way ANOVA followed by Bonferroni multiple comparison test. Compared to NA group, *p < 0.05, **p < 0.01. Data were also compared between different ZT timepoints within same feeding regimen using one-way ANOVA, ⋆p < 0.05. NA, mice fed a normal diet ad libitum; FA, mice fed a high-fat diet ad libitum; FT, mice fed a time-restricted high-fat diet; ZT, Zeitgeber time. [file Image_1.TIFF]

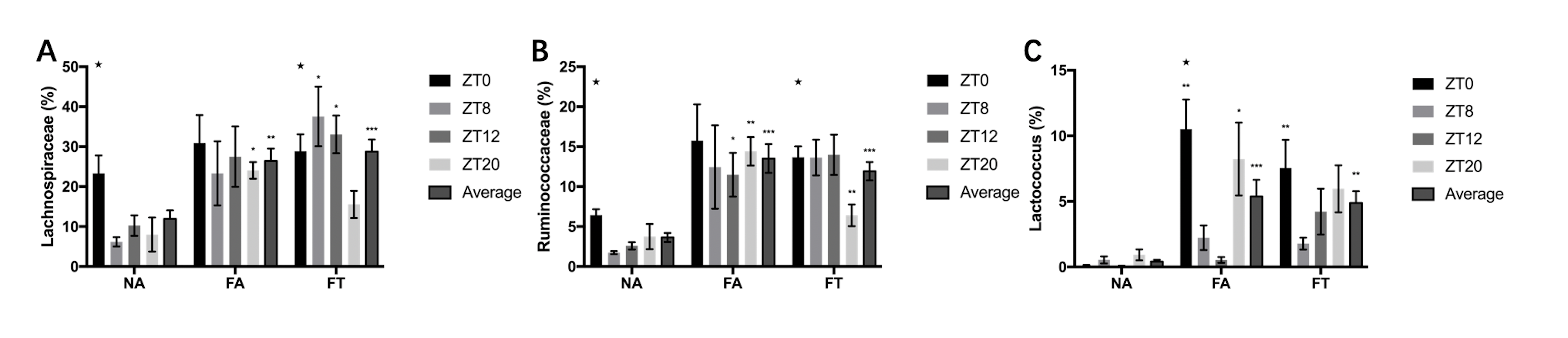

Supplement: Additional File S5 — Effects of feeding regimen on gut microbiome composition at different ZT timepoints (analyzed on subphylum level). Presents the relative abundance levels of 3 categories of interest. Effects of feeding regimen on gut microbiota. (A) family Lachnospiraceae. (B) family Ruminococcus. (C) genus Lactococcus. Relative abundance levels were shown as mean ± SEM (%) (n = 4–5 for each timepoint; n = 18–20 for the average). Data were compared between groups at same ZT timepoints using one-way ANOVA followed by Bonferroni multiple comparison test. Compared to NA group, *p < 0.05, **p < 0.01, ***p < 0.001. Data were also compared between different ZT timepoints within same feeding regimen using one-way ANOVA, ⋆p < 0.05. NA, mice fed a normal diet ad libitum; FA, mice fed a high-fat diet ad libitum; FT, mice fed a time-restricted high-fat diet; ZT, Zeitgeber time. [file Image_2.TIFF]
