# Supplementary material for: Time-Restricted Feeding Reduces the Detrimental Effects of a High-Fat Diet, Possibly by Modulating the Circadian Rhythm of Hepatic Lipid Metabolism and Gut Microbiota
Source: Front Nutr. 2020 Dec 1;7:596285. doi: 10.3389/fnut.2020.596285 (PMC7793950; doi:10.3389/fnut.2020.596285)
Supplement: Additional File S4 — Effects of feeding regimen on gut microbiota composition summarizes the abundance levels of 4 predominant phyla in gut microbiota of the three groups. [file Table_3.DOCX]

Additional file 4. Effects of feeding regimen on gut microbiota composition

|  | *Bacteroidetes* | *Firmicutes* | *Actinobacteria* | *Proteobacteria* |
| --- | --- | --- | --- | --- |
| NA | 61.34±12.99 | 34.10±13.49 | 0.944±0.829 | 2.335±1.375 |
| FA | 27.02±13.06 ^**^ | 58.04±9.33 ^**^ | 1.365±1.229 | 10.81±11.23 ^**^ |
| FT | 39.28±17.08 ^**, #^ | 47.89±12.86 ^**, #^ | 0.776±0.507 | 9.471±5.918 ^**^ |
| *p* value | <0.001 | <0.001 | 0.115 | 0.001 |

Relative abundances of data were shown as mean ± SD. n=18-20 for each group. Data were analyzed using one-way ANOVA followed by Bonferroni multiple comparison test. NA, mice fed a normal diet ad libitum; FA, mice fed a high-fat diet ad libitum; FT, mice fed a time-restricted high-fat diet. Compared to the NA group, **p*<0.05, ***p*<0.01. Compared to FA group, #*p*<0.05.
